# Arousal and Executive Alterations in Attention Deficit Hyperactivity Disorder (ADHD)

**DOI:** 10.3389/fpsyg.2020.01991

**Published:** 2020-08-12

**Authors:** Diana Martella, Nerea Aldunate, Luis J. Fuentes, Noelia Sánchez-Pérez

**Affiliations:** ^1^Facultad de Ciencias Sociales y Humanidades, Instituto de Estudios Sociales y Humanísticos, Universidad Autónoma de Chile, Santiago, Chile; ^2^Escuela de Psicología, Pontificia Universidad Católica de Chile, Santiago, Chile; ^3^Departamento de Psicología Básica y Metodología, Facultad de Psicología, Universidad de Murcia, Murcia, Spain; ^4^Departamento de Psicología y Sociología, Facultad de Ciencias Sociales y Humanas, Universidad de Zaragoza, Teruel, Spain

**Keywords:** attention deficit hyperactivity disorder, arousal, executive functions, inhibition, cognitive-energetic model

## Abstract

Attention deficit hyperactivity disorder (ADHD) is the most common neurobehavioral disorder in childhood and can significantly affect a child’s personal and social development and academic achievement. Taking into account the model of attentional networks proposed by Posner et al., the aim of the present study was to review the literature regarding two main explicative models of ADHD, i.e., the inhibition model and the cognitive-energetic model, by discussing behavioral and neurological evidence of both models and the limitations of each model. The review highlights evidence that favors the energetic model and points to an unstable arousal as a potential pathogenetic factor in ADHD.

## Introduction

Attention deficit hyperactivity disorder (ADHD) is one of the most prevalent psychiatric disorders (Polanczyk et al., 2014). ADHD is mainly characterized by varying degrees of inattention, hyperactivity, and impulsivity (American Psychiatric Association, 2013) as critical features of the disorder, which are usually accompanied by other concomitant deficits, such as lower emotional/motivational management, impaired fine motor coordination, low management of time, more frequent disruptive behavior, unstable sleep habits, academic achievement lower than their possible intellectual abilities, and impaired quality of life (Fenollar-Cortés and Fuentes, 2016). These symptoms appear at early stages of children’s development before the age of 12 (American Psychiatric Association, 2013) and may change over time (American Psychiatric Association, 2013), such as decrease in hyperactivity–impulsivity symptoms with age (Döpfner et al., 2015). However, the disorder has been found to be relatively stable and persistent from childhood to adulthood (Barkley, 2000), and it can become a chronic condition that requires social and financial support (Pelham et al., 2020). Many studies have consistently reported the negative impact of ADHD symptoms on children’s academic achievement and socioemotional development. For instance, children with ADHD exhibit impairments in acquiring mathematical and language skills compared to their peers with typical development (Kim and Kaiser, 2000; Bruce et al., 2006; McConaughy et al., 2011), problems in their interpersonal relationships with both teachers and peers (Becker et al., 2006; Coghill et al., 2006; Nijmeijer et al., 2008), and difficulties in social cognition (Uekermann et al., 2010). Additionally, children with ADHD are more likely to report depression and anxiety symptoms (Meinzer et al., 2014; Sciberras et al., 2014).

Although the first case description of ADHD was reported in 1902 (Still, 1902), the etiology, diagnosis, and treatment of ADHD are still unclear. Concerning diagnosis, the symptoms of ADHD lack significant specificity, making it difficult to detect the disease at an early stage, and the diagnosis is still mainly based on observation and informant reports (Drechsler et al., 2020). A lack of specificity in the diagnosis increases the possibility that patients may not be correctly diagnosed (Levelink et al., 2020). In addition, manifestations of ADHD seem to be very heterogeneous, and it is difficult to understand both the neurological and cognitive mechanisms underlying ADHD-related deficits (Drechsler et al., 2020; Sutcubasi et al., 2020). In a recent systematic review, Bellato et al. (2020) suggest a possible link between a dysregulation of arousal (related to the autonomic nervous system) and a deficit in attentional and executive functions as the core problem associated with ADHD. In this line, the attentional network model proposed by Posner et al. (2019) can be deemed as an appropriate theoretical framework to account for ADHD dysfunctions (Berger and Posner, 2000; Posner et al., 2019). From a cognitive neuroscience approach, the model proposes that attention is an organic system that comprises a variety of neural processes (Posner and Petersen, 1990; Fan et al., 2005). According to Posner and Petersen (1990), three specialized attentional neural networks are strictly linked with the activity of specific neuromodulator systems subtending three different attentional functions: (1) alerting – defined as achieving (phasic alerting) and maintaining (tonic alerting or vigilance) a general state of activation (or arousal) of the cognitive system, (2) orienting – defined as selectively allocating the attentional focus to potentially relevant locations/objects in the visual field, and (3) executive control – defined as the ability to control our own behavior to achieve intended goals, resolve conflict among alternative responses, and inhibit impulsive responses.

The alerting network has been associated with the right frontal and parietal lobe, and the locus coeruleus, which provides the system with norepinephrine (NE) (Cohen et al., 1987; Berger and Posner, 2000; Sturm and Willmes, 2001). The orienting network is thought to be mediated by a lateralized network located in the right superior parietal cortex and in the right inferior frontal gyrus (Corbetta and Shulman, 2002), and it is modulated by the cholinergic system (Berger and Posner, 2000; Thiel et al., 2004). Finally, the executive network is associated with the anterior cingulate cortex, the basal ganglia, and the lateral prefrontal cortex (Berger and Posner, 2000), and this network is modulated by the neurotransmitter dopamine (DA). Although deficits in the executive network have been associated with ADHD symptoms, there is also empirical evidence of ADHD patients having difficulties in controlling their level of arousal depending on the alerting network (Sergeant, 2005; Bellato et al., 2020). Given the relevance of the attentional networks functioning to characterize attention-dependent deficits in ADHD, in this review, we want to go further and present recent evidence that highlights how this particular theoretical approach may help with the diagnosis and therapeutic strategies to tackle some core deficits related to the disease. In doing so, here we adopt the cognitive neuroscience approach as a theoretical umbrella to examine two more standard models that come from a clinical neuropsychological tradition: one model emphasizes executive function deficits in ADHD that are mainly related to a failure in inhibition, and the other model emphasizes a deficit of energetic factors among ADHD patients, leading to both inattention and hyperactivity symptoms.

## Theoretical Models of Attention Deficit Hyperactivity Disorder

As mentioned previously, there are several theories to explain the ADHD symptoms, but so far, two models have been dominant in ADHD research: (1) *the inhibition model* (Barkley, 1997), which suggests that the core deficit in individuals with ADHD is poor behavioral inhibition associated with the executive network of the attention system and (2) *the cognitive-energetic model* (CEM; Sergeant, 2000), which proposes that the core problem in ADHD symptoms is a deficit in the energetic maintenance related to the alerting network. In this article, we aim to review some relevant evidence related to the two aforementioned ADHD models, and we use the Posner’s cognitive neuroscience framework of attention to discuss empirical support for and the limitations of each model. A summary of evidence favoring each model is presented in Supplementary Table 1.

### The Inhibition Model of Attention Deficit Hyperactivity Disorder

Barkley’s model (Barkley, 1997) proposes that the central deficit in ADHD is poor response inhibition, which involves three interrelated processes: (1) to inhibit the initial prepotent response to an event; (2) to stop an ongoing response, allowing a delay in the decision to reply; and (3) to control interference from distracting information (to preserve a period of delay from distracting stimuli). The dysfunction in inhibition would lead to consequences on the efficient performance of the neuropsychological abilities named executive functions (EFs) [e.g., non-verbal working memory (WM), self-regulation of affect, motivation, arousal, internalization speech or verbal WM, and reconstitution], which in turn might affect motor control. Although the definition of EF varies, most of the abilities included by Barkley have been conceptualized as part of the executive attentional network (Berger and Posner, 2000). Following this approach, if behavioral inhibition is the core deficit in ADHD, we would expect children with ADHD to perform poorly on inhibition and EF tasks compared to typically developed peers; we would also expect abnormalities in the structure and function of brain areas involved in the executive attentional network, i.e., the anterior cingulate cortex and the prefrontal cortex modulated by DA.

#### Behavioral Evidence

According to Barkley’s (1997) model, previous behavioral studies have shown that children with ADHD symptoms show some deficits in inhibition (Berlin et al., 2004; Geurts et al., 2005; Wodka et al., 2007; Schoemaker et al., 2012; see also the following meta-analyses: Oosterlaan et al., 1998; Willcutt et al., 2005; Wright et al., 2014), verbal WM (Berlin et al., 2004; Brocki et al., 2008; Kasper et al., 2012; Martinussen et al., 2005), non-verbal WM (Berlin et al., 2004; Westerberg et al., 2004; Martinussen et al., 2005; Re et al., 2010; Sowerby et al., 2011; Kasper et al., 2012), self-regulation (Braaten and Rosén, 2000; Berlin et al., 2004; Crundwell, 2005), and reconstitution (Berlin et al., 2004; Harrier and DeOrnellas, 2005). These findings might indicate that behavioral inhibition is the dysfunction underlying the ADHD symptoms. However, not all the evidence has been consistent. For instance, there are also studies that have failed to find significant differences in non-verbal WM (Berlin et al., 2004; Geurts et al., 2005; Brocki et al., 2008; Schoemaker et al., 2012), verbal WM (Sowerby et al., 2011; Pineau et al., 2019), self-regulation of arousal (Stevens et al., 2002), and reconstitution (Geurts et al., 2005) between ADHD children and controls. In those cases, researchers have stressed the importance of taking into account the ADHD subtype (individuals with the prevalent inattentive subtype might exhibit impairments in processing speed and focused attention rather than behavioral inhibition and working memory deficits; Barkley, 1997) and the developmental changes in EF (younger ADHD children, but not older children, were found to have deficits in verbal WM) to explain the inconsistent results.

In addition to the incongruent results in EF, there are also studies that have failed to find significant group differences in inhibition (Westerberg et al., 2004; Shaw et al., 2005; Brocki et al., 2008; Bioulac et al., 2014), which is especially critical because this mechanism has been proposed by Barkley to be the core failure in ADHD. To account for failures to find inhibition deficits in ADHD, some authors have examined the reliability of certain inhibition tasks, as some measures have been deemed insensitive to assess interference control in individuals with ADHD (Brocki et al., 2008). In contrast, other researchers have claimed that the reason for the non-significant results might be the underaroused state of individuals with ADHD. Similarly, Shaw et al. (2005) argued that the non-significant differences in inhibition skills between individuals with ADHD and controls, measured by computerized tasks, might be due to the greater motivation, effort, and arousal evoked by computer games than the effort induced by traditional laboratory-based tasks. Consequently, the primary deficit might not be based on response inhibition but on the difficulty experienced by individuals with ADHD in reaching an optimal level of activation.

#### Neurological Evidence

Regarding neurological evidence, we would expect individuals with ADHD to show abnormalities in the structure and function of brain areas involved in the executive attentional network (anterior cingulate, prefrontal cortex, and basal ganglia) as well as in the dopaminergic system compared to typically developed peers. In a recent review published by Gallo and Posner (2016), the authors describe the volumetric reduction found in the basal ganglia in individuals with ADHD compared to controls (Ellison-Wright et al., 2008; Nakao et al., 2011; Frodl and Skokauskas, 2012), although the effect was attenuated over time and was no longer detectable in adulthood (Nakao et al., 2011; Frodl and Skokauskas, 2012). Furthermore, some meta-analyses have shown hypoactivation in frontostriatal regions (right inferior frontal cortex, striatum, and supplemental motor cortex) (Hart et al., 2013) and frontoparietal areas (dorsolateral prefrontal, anterior cingulate, and inferior parietal cortices) in children with ADHD compared to controls during the performance of inhibition tasks (Cortese et al., 2012). According to this view, an ample medical treatment used with individuals with ADHD involves stimulant drugs targeted to counteract a hypodopaminergic state (Arnsten, 2006). Interestingly, the serendipitous discovery that amphetamines make children calmer and more focused (Glaser and Gerhardt, 2012) has strongly supported the hypodopaminergic ADHD hypothesis. In addition, whereas the prevalence of DA in the nucleus accumbens may explain the ADHD alterations in reward sensitivity, the low levels of DA in the striatum may justify ADHD hyperactivity, and the presence of DA in the frontal cortex may support the decreased inhibitory control in ADHD (Berridge and Devilbiss, 2011).

### The Cognitive-Energetic Model of Attention Deficit Hyperactivity Disorder

The CEM focuses on energetic factors as the most critical explanation of ADHD, proposing that the deficit of these factors leads to both inattention and hyperactivity symptoms. This model claims that the overall efficiency of information processing is determined by the interplay among three mechanisms at different levels, with top-down and bottom-up streams among them (Sergeant, 2000). The *lower level* includes four general stages of computational mechanisms of attention: encoding, search, decision, and motor organization. The *middle level* comprises the energetic pools: arousal, effort, and activation. The arousal pool is defined as a phasic response that is time-locked to stimulus processing and is typically influenced by signal intensity and novelty, and it is behaviorally indexed by sleep–wake patterns. The effort pool is characterized by the energy necessary to meet with task demands, is located within the hippocampus, and seems to function by both exciting and inhibiting the other two energetic pools (arousal and activation). The activation pool is defined as the tonic physiological readiness to respond. Finally, *the upper level* refers to the executive control system and is associated with planning, response inhibition, error detection and correction responses, WM, and flexibility, among other abilities.

#### Behavioral Evidence

Following this approach, impairments observed in individuals with ADHD are expected at the three levels of the CEM. For instance, motor difficulties have been reported in children with ADHD (Goulardins et al., 2013; Kaiser et al., 2015; Rosa Neto et al., 2015; Fenollar-Cortés and Fuentes, 2016; Fenollar-Cortés et al., 2017), which might reflect dysfunction at the lower level. Moreover, children with ADHD generally exhibit a dysfunction in their response inhibition (upper level), as we mentioned previously. However, this dysfunction is not specific to ADHD, as this deficit is also common to other disorders, such as conduct disorder and autism spectrum disorders (Oosterlaan et al., 1998; Geurts et al., 2014). In fact, evidence suggests that inhibition deficits associated with ADHD may, at least in part, be due to energetic dysfunction (Sergeant et al., 1999). Although there are few specific tests to directly measure the energetic pool, some studies show that the event rate, that is, the speed with which stimuli are presented, affects the energetic state, and that the event rate is an important determinant of performance (Sergeant, 2005). A slow event rate would induce a rather underarousal/underactivation state, with slow and inaccurate responses, whereas a fast event rate would lead to a rather overarousal/overactivation state, resulting in fast and inaccurate responses. In fact, some behavioral studies indicate that individuals with ADHD perform more poorly in tasks involving slow event rates, with a significant slowing in reaction times (Meere et al., 1992; Wiersema et al., 2005, 2006; for a review, see Metin et al., 2012), although the results are not consistent (e.g., Raymaekers et al., 2007).

#### Neurological Evidence

The conception of ADHD as a result of a hypoaroused brain state has been supported by electrophysiological studies. Previous electroencephalographic (EEG) studies among children with ADHD have reported an increase in low-frequency power (predominantly in the theta band) and a decrease in high-frequency power (alpha and beta bands) (Kuperman et al., 1996; Clarke et al., 2001, 2002; Markovska-Simoska and Pop-Jordanova, 2017; Clarke et al., 2019). Specifically, the theta band has been related to low activation, such as in drowsiness states and relaxed wakefulness (Scher, 2017; Mari-Acevedo et al., 2019), whereas alpha and beta bands have been associated with goal-directed activities, sustained attention, and higher task-related attention (Laufs et al., 2006; Dockree et al., 2007). A greater ratio of the theta power band to the beta band (increased theta/beta ratio index) may reflect a reduced cortical control function, resulting in motivational imbalances (Schutter and Van Honk, 2005). Moreover, this index has been found to be strongly associated with ADHD symptoms (Barry et al., 2003; Jarrett et al., 2017; Markovska-Simoska and Pop-Jordanova, 2017). In addition, evidence suggests that this difference in theta/beta ratio decreases across years (Arns et al., 2013; Markovska-Simoska and Pop-Jordanova, 2017). This hypoaroused cortical state has been mainly localized in the frontal and posterior areas (Clarke et al., 2002), the same brain regions that have been identified as the neuroanatomical substrates of the attentional network (Posner, 2012). Since the right hemisphere (RH) plays a crucial role in maintaining and controlling both arousal and the intensity aspects of attention (Sturm and Willmes, 2001), if the pathophysiology of ADHD was a deficit in the arousal system, we would expect people with ADHD to show RH dysfunction. In fact, several studies have highlighted an impairment in both anterior and posterior RH areas related to the attentional system in ADHD (Stefanatos and Wasserstein, 2006). These dysfunctions are further supported by both behavioral and neuroimaging data (van Ewijk et al., 2012).

The alerting system proposed by Posner and Petersen (1990) has been related to the noradrenergic system and may modulate attention and arousal. Many studies have shown different neurotransmitters implied in ADHD, with NE being the most important neurotransmitter, and increasing evidence has shown less NE in ADHD patients than in controls (i.e., Biederman and Spencer, 1999). Since ADHD symptoms involve both attentional and arousal deficits, it is plausible that dysregulation of the NE system may be involved in the pathophysiology of ADHD (Biederman and Spencer, 1999; Park et al., 2012). Furthermore, the atomoxetine, a drug effectively used for ADHD treatment, acts as an inhibitor highly selective of noradrenergic reuptake (Arnsten, 2006), which strongly supports a relationship between NE and ADHD. Finally, the depletion of NE results in increased distractibility and motor hyperactivity, both typically ADHD symptoms. In contrast, stimulation of the NE system is associated with a decrease in distractibility and an improvement of cognitive functions (Sengupta et al., 2012).

## Conclusion

The exact etiopathogenesis of ADHD is still unclear. In this review, we discuss the behavioral and neurological previsions and evidence of two well-known models: *the inhibition model*, proposed by Barkley (1997), and *the cognitive-energetic model*, introduced by Sergeant (2000). On the one hand, the hypothesis of a dysfunction of the DA system and the related Barkley’s model do not seem to explain all the characteristic symptoms of ADHD. Inconsistent results (e.g., Brocki et al., 2008; Bioulac et al., 2014; Pineau et al., 2019) highlight the relevance of considering other factors, such as the ADHD subtype, as well as the maturation of executive functions throughout life. On the other hand, many results (e.g., Metin et al., 2012; Clarke et al., 2019; Bellato et al., 2020) seem to support the hypothesis of dysfunction of the locus coeruleus-norepinephrine (LC-NE) system and therefore to sustain the hypoarousal and the RH hypothesis in addition to Sergeant’s energetic model. In line with this, it has recently been proposed that the core problem in ADHD is an unstable arousal (Hegerl and Hensch, 2014), and numerous findings seem to support this hypothesis (e.g., Strauß et al., 2018; Bellato et al., 2020). From this point of view, hyperactivity and sensation seeking are considered an organism’s regulatory response to an unstable level of cortical arousal. The model provides an explanation for the attentional deficits, especially related to sustained attention and vigilance, and for the ADHD subtypes (Hegerl and Hensch, 2014). Furthermore, sleep problems and circadian alterations are very common in ADHD patients, and many studies (e.g., Yoon et al., 2012; Bioulac et al., 2020) have indicated that all factors inducing sleep deficits and dysregulation of arousal can impair ADHD symptoms. Finally, drug stimulants are known to reduce slow wave activity, attention deficits, and hyperactivity through the regularization of the arousal level. All of this evidence points to the dysregulation of arousal as a biomarker of ADHD, a relevant factor that should be considered in diagnostic processes and intervention programs.

## Author Contributions

NA and DM conceived this manuscript. NA and DM reviewed the literature. NS-P and DM wrote the first draft of the manuscript. DM, NA, and LF edited and provided intellectual input to the manuscript. All authors approved the final version of the manuscript for submission.

## Conflict of Interest

The authors declare that the research was conducted in the absence of any commercial or financial relationships that could be construed as a potential conflict of interest.
