# Supplementary material for: Arousal and Executive Alterations in Attention Deficit Hyperactivity Disorder (ADHD)
Source: Front Psychol. 2020 Aug 12;11:1991. doi: 10.3389/fpsyg.2020.01991 (PMC7434859; doi:10.3389/fpsyg.2020.01991)
Supplement: Supplementary file 1 [file Table_1.pdf]

Summary of principal evidences for each theoretical model (systematic reviews, meta-analysis studies and original researches only are reported).

Source	Type of study	Target group	Measure	Outcome measure	Summary of findings	Model
**Arns et al., 2013**	Meta-analysis	6 to 18 years (n=1770). ADHD group (n=1253), control group (n=517)	EEG	Theta/Beta ratio	The elevated Theta/Beta ratio is found in a substantial ADHD group (25%-40%), demonstrating a prognostic value of this measure in treatment outcomes, rather than a diagnostic measure.	Cognitive-Energetic
**Bellato et al., 2020**	Systematic review	Children, adolescents and adults (unspecified age)<br><br>(55 studies)	Heart rate, Electrodermal-activity, Pupillometry	Resting state measures and cognitive tasks	Results indicated a dysfunction in the autonomic nervous system in ADHD patients, especially related to hypo-arousal.	Cognitive-Energetic
**Berlin et al., 2004**	Original research<br><br>(quasi-experimental study)	7 to 10 years (n=63). ADHD group (n=21), control group (n=42)	(1) go/no-go task, (2) Stroop-like task, (3) time reproduction, (4) hand movements, (5) continuous performance task, (6) puzzle cheating task and (7) story reconstitution	(1) inhibition (commission and omission errors), (2) interference control (errors) (3) working memory (errors), (4) WM (errors), (5) self-regulation of arousal (omission errors and parental rating scale, (6) internalization of speech (scale of poor rule-governed behavior), (7) story reconstitution after a delay (information units)	ADHD children showed a poorer performance with regard to controllers in all measures, except repetition of hand movements. In logistic regression models, interference control, time reproduction, and emotion regulation were shown to be significant independent predictors of group membership.	Inhibition
**Bioulac et al., 2014**	Original research<br><br>(quasi-	Unspecified age (n=48). ADHD group	(1) Continuous Performance Test II, and three commercial	All measures assessed inhibition ability, in terms of (1) number of	ADHD children had lower performance on the CPT II than controls, but equivalent	Inhibition

	experimental study)	(n=26), control group (n=16)	videogames: (2) Secret Agent, (3) Bubble Pop, and (4) Kung 2	omission errors, (2) collected objects and total score, (3) number of burst bubbles and total score, (4) total score	performances to controls when playing video games	
**Braaten&Rosén, 2000**	Original research<br><br>(quasi-experimental study)	6.00 to 12.81 years (n=45). ADHD group (n=24), control group (n=19)	(1) Empathy response task, (2) Emotions Behavior Checklist, (3) Emotional Intensity Scale for Children, (4) Emotional Reactions to External Contingencies Scale for Children	(1) Empathy response assessed by match score (similarity between character's and participant's emotion) and an interpretation score (from egocentric to character-centered interpretation), (2) Emotional behaviors (3) Emotional intensity, (4) Emotional reactions to contingencies	ADHD boys were less empathic than boys without ADHD, and exhibited more behavioral manifestations of sadness, anger, and guilt than did boys without ADHD. No differences were found on measures of emotional intensity or emotional reactions to external contingencies	Inhibition
**Brocki et al., 2008**	Original research<br><br>(quasi-experimental study)	7 to 12 years old (n=65). ADHD group (n=31), control group (n=34)	(1) Letter–number sequencing subtest, (2) pig house task, (3) the visuospatial WM game, (4) self-ordered pointing task, (5) Stroop Color–Word Task, (6) inhibitory conflict task	(1) verbal WM (correct responses), (2) visuospatial WM (correct responses), (3) visuospatial WM (number of errors), (4) visuospatial WM (number of errors), (5) interference control, (6) inhibitory control (percentage of errors)	ADHD boys showed a significant weakness in verbal and visuospatial WM, and poorer performance on prepotent response inhibition compared to control group. Logistic regression analyses showed that: (a) WM together with the inhibitory conflict tasks differentiated between children with ADHD and controls, (b) deficits in WM contributed with variance over and above that of prepotent response inhibition in predicting group membership, whereas prepotent response inhibition contributed with variance that was not independent of WM.	Inhibition

**Clarke et al., 2001**	Original research<br><br>(quasi-experimental study)	8 to 12 years (n=80). Three groups: boys and girls with ADHD, and controls	EEG resting state	Total power, relative alpha, theta/alpha and theta/beta ratios	Results indicated an elevated EEG slow wave activity in ADHD children compared to controls, primarily in the theta band, suggesting a dysfunction in the arousal system	Cognitive-Energetic
**Clarke et al., 2002**	Original research<br><br>(quasi-experimental study)	8 to 12 years. Three groups: 2 ADHD groups and controls (20 males in each group)	EEG resting state	Total and relative power of alpha, beta, theta, delta, theta/alpha and theta/beta ratios	Results showed more absolute and relative EEG slow wave activity (theta and delta waves) in ADHD children compared to controls, confirming the hypo-arousal hypothesis	Cognitive-Energetic
**Clarke et al., 2019**	Origianal research<br><br>(longitudinal study)	Children and Adults (n=75 ;25 ADHD, and two control groups)	EEG resting state	Relative power of alpha, beta, theta, delta	Results indicated a significant EEG maturation with increasing age, particularly as respect to the elevated EEG slow wave activity present in childhood, suggesting a change in the arousal mechanisms	Cognitive-Energetic
**Cortese et al., 2012**	Meta-analysis article	Fifty-five studies (39 with children and 16 with adults)	Task-based (inhibition, WM, and vigilance/ attention) functional MRI studies	Activation of brain networks: frontoparietal, dorsal attention, somatomotor, visual and default networks	Results with children' studies: hypoactivation in ADHD relative to comparison subjects was observed in systems involved in executive function (frontoparietal network) and attention (ventral attentional network), whereas hyperactivation was observed in the default, ventral attention, and somatomotor networks. In adults, ADHD-related hypoactivation was predominant in the	Inhibition

					frontoparietal system, while ADHD-related hyperactivation was present in the visual, dorsal attention, and default networks.	
**Crundwell, 2005**	Original research (quasi-experimental study)	6 to 11 years (n=45). ADHD group (n=24), control group (n=19)	(1) Children's Behavior Questionnaire, (2) Self-Control Rating Scale, (3) the Affect Intensity Scale, (4) Child Behavior Inventory, (5) Disruptive Behavior Rating Scale	(1) regulation (impulsivity and inhibitory control scores), and emotionality (anger/frustration and sadness scales), (2) regulation (children's ability to inhibit negative behavior), (3) emotionality (frequency and intensity of emotional states), (4) behavior (frequency of behavior problems), and (5) ADHD symptoms (frequency of ADHD symptoms).	ADHD boys who showed greater anger/frustration and less self-control and inhibition were rated as experiencing more behavioral difficulties. Children with symptoms of hyperactivity and impulsivity were rated as lower in self-control and inhibition and higher in emotionality.	Inhibition
**Ellison-Wright et al., 2008**	Meta-analysis	11 studies	Voxel-based morphometry analysis of MRI datasets	Gray matter density differences in whole-brain	There is gray matter reduction in the right putamen/globus pallidus region for people diagnosed with ADHD, which authors suggested to be an anatomical marker for dysfunction in frontostriatal circuits mediating cognitive control	Inhibition
**Frodl&Skokauskas, 2012**	Meta-analysis	11 voxel-based morphometry studies, and 7 studies having used a manual tracing	MRI voxel-based morphometry and manual tracing studies	Gray matter volumes	There was a reduced right globus pallidus, putamen, and caudate volumes in children with ADHD. Adult patients with ADHD showed volume reduction in the anterior	Inhibition

					cingulate cortex	
**Gallo & Posner, 2016**	Review article					Inhibition
**Geurts et al., 2005**	Meta-analysis	41 studies	Prepotent response inhibition and interference control tasks	Inhibitory control	Individuals with ASD showed increased difficulties in prepotent response inhibition and in interference control	Inhibition
**Glaser & Gerhardt, 2012**	Review article					Inhibition
**Goulardins et al., 2013**	Original research<br><br>(cross-sectional study)	7 to11 years (n=66). ADHD group (n=34), control group (n=32)	Motor Developmental Scale	Global motricity, balance, body scheme, spatial and temporal organization measures	The motor profile showed a significant poor motor performance in children with ADHD as respect to controls, which might reflect a dysfunction at the lower level proposed in the Sergeant hypothesis	Cognitive-Energetic
**Harrier &DeOrnellas, 2005**	Original research<br><br>(quasi-experimental study)	8 to 12 years (n=177). ADHD group (n=93), control group (n=85)	(1) Planning task (the Woodcock Johnson-III of Cognitive Abilities), (2) Wechsler Mazes task (WISC-III), (3) Elithorn Mazes task (WISC-III PI), (4) Analysis Synthesis and Concept Formation tests	(1) Planning (tracing a pattern without lifting the pencil), (2) Planning (drawing a line from the center of a maze to the outside without crossing lines), (3) Planning (tracing a line following the rules and time limit), and (4) Reconstruction (deductive and inductive reasoning).	Inattentive and Combined ADHD had significantly lower scores on the measure of reconstruction when compared to the control group.	Inhibition
**Hart et al., 2013**	Meta-analysis	21 data sets were included for inhibition, and 13 data sets	Functional Magnetic Resonance imaging studies during inhibition and	Brain activation in different structures	Compared to controls, ADHD patients showed reduced activation for inhibition in the right inferior frontal cortex,	Inhibition

		were included for attention	attention tasks		supplementary motor area, anterior cingulate cortex, and striato-thalamic areas. In the case of attention, ADHD group showed reduced activation in the right dorsolateral prefrontal cortex, posterior basal ganglia, and thalamic and parietal regions.	
**Jarrett et al., 2017**	Original research (quasi-experimental study)	7 to 12 years (n=40). ADHD group (n=21), control group (n=20)	Neuropsychological assessment, EEG resting state	Theta and beta power, Theta/beta ratio	Significant differences (particularly for theta/beta ratio in frontal and frontocentral regions) were found in ADHD children as respect to controls.	Cognitive-Energetic
**Kaiser et al., 2015**	Systematic Review	6 to 13 years (n=3649). ADHD group(n=1887), medicated ADHD group (n=344); control group (n=1418)	Motor tests and batteries	Characterization of motor response (e.g., reaction time, response initiation time, etc.)	Children with ADHD present frequently motor skill deficits, confirming a dysfunction at the lower level proposed in the Cognitive-Energetic Model	Cognitive-Energetic
**Kasper et al., 2012**	Meta-analysis	45 studies	Phonological and visuospatial storage/rehearsal tasks	Phonological and visuospatial storage/rehearsal subsystems of WM	Children with ADHD exhibit significant WM deficits compared to their typically developing peers	Inhibition
**Kuperman et al., 1996**	Original research (quasi-experimental study)	7 to 13 years (n=30). ADHD group (n=16), undifferentiated attention deficit disorder group (n=12), disruptive	Spectral EEG, Event Related Potentials (ERP)	Spectral power of delta and beta bands; latency and amplitude of N1 and P3 components	ADHD children showed an increased beta band percent power, un increase in the N1 latency and a decrease amplitude in the P3. These results are in line with a dysfunction in arousal and	Cognitive-Energetic

		disorder group (n=12)			attentional networks	
**Markovska-Simoska & Pop-Jordanova, 2017)**	Original research<br><br>(quasi-experimental study)	9 mean age and 35.8 mean age (n=60). ADHD boys' group (n=30), male adults' group (n=30)	EEG resting state	Absolute and relative EEG power of delta, theta, alpha y beta bands. Theta/beta ratio	ADHD children showed an increased absolute power of slow waves (theta and delta), as respect to adults and greater relative power than the adults in the delta and theta bands. Results suggests a difference in cortical arousal between children and adults and in maturation processes	Cognitive-Energetic
**Martinussen et al., 2005**	Meta-analysis	26 studies	Verbal and spatial WM tasks	Verbal and spatial WM and type of processing required (storage versus storage/manipulation)	ADHD children showed deficits in spatial storage, spatial central executive WM, verbal storage and verbal central executive WM, independently of comorbidity with language learning disorders and IQ, compared to control children.	Inhibition
**Meere et al., 1992**	Original research<br><br>(quasi-experimental study)	104 to 144 months (n=29). Hyperactive group (n=9), control group (n=20)	Choice reaction time task	Reaction time, percentage of errors, omissions, reaction times during catch trials	Hyperactive children showed difficulties in motor processes prior to target presentation as respect to controls, confirming a dysfunction at the lower level proposed in the Cognitive-Energetic Model	Cognitive-Energetic
**Metin et al., 2012**	Meta-analysis	30studies,<br><br>Unspecified sample size (mean age=11)	Go/NoGo task	Reaction time, event rate effect	Results indicated slowing of reaction time in ADHD relative to controls on trials with slow event rates, in line with the state regulation deficit model of ADHD	Cognitive-Energetic

**Nakao et al., 2011**	Meta-analysis	14 data sets	Voxel-based morphometry studies	Gray matter volumes in brain regions	ADHD patients had greater gray matter volumes in the left posterior cingulate cortex, and global reductions in gray matter volumes, localized in the right lentiform nucleus and extended to the caudate nucleus, compared to control groups.	Inhibition
**Oosterlaan et al., 1998**	Meta-analysis	8 studies	(1) Mean reaction time, (2) the inhibition function (IF), (3) Stop signal reaction time, (4) the slope of the IF plotted as a function of ZRFT	Response inhibition	Results revealed a consistent evidence for a response inhibition deficit in ADHD, but this deficit did not distinguish children with ADHD from children with conduct disorder, nor from children with comorbid ADHD+conduct disorder	Inhibition
**Park et al., 2012**	Original research<br><br>(pre-post treatment)	8.9mean age (n=39)	Clinical assessment, genomic DNA extraction, baseline Single-photon emission computed tomography (SPECT)	SPECT standard templates	Results indicated no differences in baseline clinical assessments or cerebral perfusion based on genotype. After treatment, ADHD children with the G/G genotype showed an improvement in symptoms as respect to the other ADHD children, indicating a contribution of norepinephrine transporter gene in the phenotype of ADHD.	Cognitive-Energetic
**Pineau et al., 2019**	Original research<br><br>(quasi-experimental study)	7,8 to 12,9 years (n=61). ADHD group (n=36); control group (n=25)	(1) Functional Magnetic Resonance imaging, (2) saliva test, (3) verbal N-back task	1) brain activation, (2) genotype, (3) verbal WM	WM performance was similar for ADHD and control groups. The results indicated that DAT1 VNTR polymorphism might modulate WM-related	Inhibition

					brain activity ADHD children.	
**Re et al., 2010**	Original research<br><br>(quasi-experimental study)	5 years (n=46). ADHD group (n=23); control group (n=23)	The Dual Request Selective task	Visuospatial WM	Children with ADHD symptoms performed more poorly than controls, with more intrusion errors	Inhibition
**Rosa Neto et al., 2015**	Original research<br><br>(cross-sectional study)	5 to 10 years (n=200). ADHD group (n=50), control group (n=150)	Motor Development Scale	Fine and global motricity, balance, body schema, and spatial and temporal organization	Results showed motor difficulties and a delay in motor development in ADHD children as respect to controls, confirming a dysfunction at the lower level proposed in the Cognitive-Energetic Model	Cognitive-Energetic
**Schoemaker et al., 2012**	Original research<br><br>(quasi-experimental study)	3.5 to 5.5 years (n=202). ADHD group (n=61), disruptive behavior disorder -DBD- group (n=33), comorbid ADHD+DBD group (n=52), control group (n=56)	(1) Go/No-Go task, (2) Snack Delay tasks, (3) Shape School task, (4) Nine Boxes task, and (5) Delayed Alternation	(1) Inhibition (the proportion correct), (2) Inhibition (number of intervals following the rules divided by the total number of intervals), (3) Inhibition (number of correct responses divided by the total number trials), (4) WM (number of correct retrievals divided by modified the total trials), (5) WM (number of correct retrievals divided by the number of total trials)	ADHD and DBD children exhibited inhibition deficits. When IQ was controlled, differences were still significant for ADHD group, whereas for DBD group were carried mostly by the effect on the task where motivational demands were high. Deficits in inhibition in the comorbid group were also found, but were more severe than in the DBD group. Regarding WM, few group differences were found.	Inhibition
**Sengupta et al., 2012**	Original research<br><br>(family-based)	6 to 12 years (n=412). All ADHD	Conners Global Index for Teachers and Parents, and Child Behavior Checklist measures, DNA extraction	Family-based association tests (examining transmission disequilibrium of a specific allele/haplotype from parent to affected	Results suggest that haplotype blocks within different regions of SLC6A2 show differential association with the disorder based on sex and subtype.	Cognitive-Energetic

				offspring)		
**Shaw et al., 2005**	Original research<br><br>(quasi-experimental study)	6 to 14 years (n=32). ADHD group (n=16), control group (n=16)	(1) The Revenge of Frogger (a commercial game), (2) Crash Bandicoot II (a commercial game), (3) Conners' Continuous Performance Test II, (4) Pokémon Task	Inhibitory performance (1) mean number of moves, mean errors, (2) mean number of moves, mean errors, (3) commission errors, (4) commission errors.	The performance of ADHD group on commercially games is equivalent to that of typically developing participants and is significantly better on the more game-like version of the CPT II	Inhibition
**Sowerby et al., 2011**	Original research<br><br>(quasi-experimental study)	6 to 12 years (n=80). ADHD group (n=40), control group (n=40)	(1) Finger Windows test from the Wide Range Assessment of Learning and Memory, (2) the spatial span task from the Wechsler Intelligence Scale for Children, (3) Digit Span subtest from the WISC-IV, (4) the verbal WM test from the WRAML-2	(1) visuospatial WM, (2) visuospatial WM, (3) verbal WM, (4) verbal WM	ADHD children exhibited impaired visuo-spatial WM performance. ADHD children with less than 8 years old showed deficits in verbal WM.	Inhibition
**Stevens et al., 2002**	Original research<br><br>(quasi-experimental study)	7 to12 years (n=152). ADHD group (n=76), control group (n=76)	(1) Stop-signal task, (2) Digit Span Task	(1) behavioral inhibition, (2) WM (processing and storage of the stimuli), and short-term memory (only storage of the stimuli)	Children with ADHD exhibited deficits in inhibitory control, working memory, and short-term memory relative to children without the disorder. There was no evidence for the hypothesis that reinforcement would have a differential effect on response inhibition in ADHD children compared to control children.	Inhibition
**Strauß et al., 2018**	Original research	20 to 51 (n=68). ADHD	EEG15-min resting	Mean EEG-vigilance (indexing arousal level)	ADHD adults showed significantly lower arousal	Cognitive-

	(cross-sectional study)	group (n=30), control group (n=35)		and arousal stability score (indexing arousal regulation)	levels and significantly less stable brain arousal regulation than controls, supporting the arousal regulation model of ADHD	Energetic
**Westerberg et al., 2004**	Original research<br><br>(quasi-experimental study)	Unspecified age (n=23). ADHD group (n=11), control group (n=12)	(1) the visuo-spatial working memory test, (2) the choice reaction time test, (3) the Continuous Performance test, (4) Go/no-go task	(1) visuo-spatial WM (number of items correct), (2) processing speed, (3) attention, (4) inhibition	ADHD group performed poorly on the visuospatial WM test and the VSWM test (P <.01) and the choice reaction time test, compared to the control group. However, there was no significant difference on the Continuous Performance test or Go/no-go task.	Inhibition
**Wiersema et al., 2005**	Original research<br><br>(quasi-experimental study)	7 to 12 years (n=18). ADHD group (n=9), control group (n=9)	Go/no-Go task,<br><br>ERPs	Reaction time, errors of commission, P3	Children with ADHD, relative to controls, responded more slowly and more variably in the slow event rate condition, which was accompanied by a smaller P3, supporting the state regulation hypothesis of ADHD	Cognitive-Energetic
**Wiersema et al., 2006**	Original research<br><br>(experimental study)	18 to 50 years (n=38). ADHD group (n=19), control group (n=19)	Go/No-Go task and ERPs	Mean reaction time, percentage of commission and omission errors. P3 and N2 amplitude	Results indicated a deficit in state regulation and not in response inhibition in adults with ADHD as respect to control, in agreement with the Cognitive/Energetic hypothesis	Cognitive-Energetic
**Willcutt et al., 2005**	Meta-analysis	83 studies	(1) Stop-signal Reaction Time, (2) Continuous Performance Test, (3) Wisconsin Card Sorting Test, (4) Trail making Test Part B, (5) Tower of	(1) Response inhibition, (2) Response inhibition (commission and omission errors), (3) Set-shifting, (4) Set-shifting, (5) Planning, (6) Planning, (7) Planning/Organization,	ADHD groups showed significant impairment on all EF tasks.	Inhibition

			Hanoi/London, (6) Porteus Mazes, (7) Rey-Osterreith Complex Figure Test, (8) WM Sentence Span, (9) Digits Backward, (10) Self-ordered pointing, (11) CANTAB Spatial Working Memory	(8) verbal WM, (9) verbal WM, (10) spatial WM, (11) spatial WM		
**Wodka et al., 2007**	Original research<br><br>(quasi-experimental study)	7 to 16 years old (n=142). ADHD group (n=58), control group (n=84)	(1) Skeletomotor go/no-go test, (2) Cognitive go/no-go test, (3) Motivation-linked go/no-go test with reward and response cost	(1) Skeletomotor go/no-go test, (2) Cognitive response inhibition, (3) Response inhibition at the motivational level. The three tasks used the percentage of commission and omission errors, reaction time, and reaction time variability to measure the response inhibition.	Children with ADHD made significantly more errors of commission for the simple, cognitive, and motivation-linked go/no-go tests than the control group.	Inhibition
Wright et al., 2014	Meta-analysis	318 studies	(1) go/no-go task, (2) Conners' continuous performance task, (3) sustained attention to response task	Response inhibition	Small-to-medium deficits in withholding in various psychological disorders (e.g., anxiety, and bipolar disorder), which authors interpreted as that deficits in withholding are insufficiently specific to be used as a diagnostic measure or biomarker in most disorders.	Inhibition
